# Validation of AURA‐W: An AI‐Driven 3D Imaging System Metrics for Objective Wrinkle Assessment After Botulinum Toxin Treatment

**DOI:** 10.1111/jocd.70915

**Published:** 2026-06-17

**Authors:** Fabrizio Vignoli, Maurizio Cavallini, Emanuele Bartoletti, Lukas Buchmann, Gemma Taverni

**Affiliations:** ^1^ Sime Società Italiana di Medicina Estetica Rome Italy; ^2^ Centro Clinico Agorà Milan Italy; ^3^ Hexagon Aura Reality AG Zurich Switzerland

**Keywords:** 3D imaging, artificial intelligence, AURA, botulinum toxin, photogrammetry, wrinkles, wrinkles scale

## Abstract

**Background:**

Most clinical assessments of botulinum toxin rely on subjective observation, limiting reproducibility. The Aura 3D Imaging System combines high‐resolution photogrammetry imaging with AI analysis to provide standardized wrinkle measurements.

**Objective:**

Validate the Aura 3D Imaging System and the AURA‐W scale as an objective tool for assessing wrinkle reduction for botulinum toxin treatment.

**Methods:**

Standardized 3D captures were obtained before treatment and at follow‐up (about 27 days after) for 100 participants. Letibotulinum Toxin A was injected into the glabellar, forehead, and periorbital regions. The AURA‐W was used to quantify wrinkle severity across facial regions.

**Results:**

Wrinkle scores decreased consistently after treatment, with the greatest improvements in the upper face. Periocular changes were smaller but still evident.

**Conclusion:**

The AURA‐W enables objective, reproducible assessment of wrinkle reduction following botulinum toxin therapy. By integrating 3D imaging with AI analysis, it overcomes limitations of subjective scales and enhances precision in both clinical evaluation and research.

## 1 Introduction

In the past 20 years, the field of aesthetic medicine has experienced rapid growth, driven by rising patient demand, technological advancements, and an expanding range of treatment modalities. As this landscape evolves, there is a critical need for objective, reproducible, and scientifically rigorous methods to assess the efficacy of new procedures, techniques, and products. Traditional evaluation approaches—often based on subjective clinical judgment, patient‐reported outcomes, or photographic comparisons—lack consistency and standardization [1, 2].

To address this gap, we propose a measurement system that combines photogrammetry, enabling accurate and consistent three‐dimensional (3D) capture of physical features, with the analytical capabilities of Artificial Intelligence (AI) and data‐driven algorithms. This hybrid model produces high‐fidelity, quantifiable assessments that are both repeatable and scalable. The system, known as the Aura 3D Imaging System, was developed by Aura Reality AG.

The 3D system has progressively supplanted the two‐dimensional (2D) approach and even more traditional anthropometric techniques. Although conventional 2D photography is cost‐effective, it depends heavily on patient cooperation, is often time‐consuming, and provides lower accuracy compared to 3D methods [3, 4, 5, 6, 7, 8, 9]. The Aura 3D Imaging System enables fast and effortless capture of the complete facial geometry, allowing for an accurate digital representation of the patient's face. Moreover, the 3D approach offers higher precision than 2D, supports comparison across multiple dimensions, and facilitates a more comprehensive understanding of facial geometry and shape. By contrast, 2D images are easier to manipulate, which constitutes a disadvantage as it increases the risk of inaccuracies and false interpretations.

Leveraging advances in computer vision and machine learning applied to large datasets, this system establishes a standardized framework for evaluating treatment outcomes across time points, clinicians, and patient populations. It supports evidence‐based practice, enhances decision‐making, and provides a reliable benchmark for comparison.

This study focuses on the development and validation of the Aura Unified Rating Algorithm for Wrinkles (AURA‐W), a scientifically grounded metric designed to objectively quantify the aesthetic effects of botulinum toxin therapy. As botulinum toxin primarily acts by reducing facial wrinkles, one of the most visible and clinically relevant outcomes, this study concentrates on the detection, measurement, and evaluation of wrinkle patterns. By combining high‐precision photogrammetric data capture with AI‐driven analysis, the AURA‐W aims to offer a standardized, reproducible tool for wrinkle assessment applicable in both clinical and research settings.

Through this work, we aim to introduce a consistent and reliable scale that addresses the current lack of uniformity across studies and supports the advancement of outcome assessment in aesthetic medicine.

### 1.1 Scales and Metrics in Literature

Botulinum Toxin is primarily used to reduce the appearance of facial wrinkles, which are among the most visible signs of aging. Wrinkles are defined as creases, lines, or folds that develop on the skin as a result of intrinsic aging processes, including decreased skin elasticity, structural collagen degradation, and reduced moisture retention. These effects are often worsened by extrinsic factors such as sun exposure and repetitive facial muscle activity.

The clinical efficacy of Botulinum Toxin in reducing facial wrinkles has been demonstrated in numerous studies. However, across the existing literature, a variety of evaluation scales and measurement methodologies have been employed, resulting in inconsistencies that hinder comparability between studies.

Wrinkle evaluation methods in aesthetic medicine have evolved significantly over time, but they remain heterogeneous, with no universally accepted standard. Broadly, these methods can be understood as falling into four overlapping categories: Photography‐based visual scales, patient‐reported outcome measures, investigator‐based clinical assessments, and, more recently, AI‐driven evaluation systems (Table 1). Each approach contributes valuable perspectives but also introduces variability that complicates cross‐study comparisons. Moreover, none of these methods primarily focus on the objective, quantitative measurement of wrinkles themselves, further limiting the ability to achieve standardized and reproducible assessments.

**TABLE 1:** Wrinkles metric state of the art.

Type	Scale Names	References
Photography	LWS, MFWS, 4‐PPS, GLS	[1, 10, 11, 12, 13, 14, 15, 16]
Questionnaire	FLSQ, FLO‐11, FILLER‐Q, FACE‐Q	[17, 18, 19, 20]
Investigators	GAIS	[21, 22]
Gen‐AI	SCAWA	[23]
Imaging + AI	Aura Unified Rating Algorithm for Wrinkles (AURA‐W)	*This study*

Among the most widely used are visual scales based on photographic references. The Lemperle Wrinkles Scale (LWS), introduced by Lemperle et al. [10, 11], is the best known in the aesthetic community. It assigns scores ranging from 0 to 5 to different facial regions using reference images. The original paper correlated these scores with physical wrinkle depth, measured via silicone impressions. Building on this concept, the Modified Fitzpatrick Wrinkle Scale (MFWS) proposed by Shoshani et al. [12, 13] simplifies wrinkle severity into three main categories, each defined by representative photographs and accompanying descriptions. A similar visual assessment method is the 4‐Point Photographic Evaluation Scale (4‐PPS), which evaluates wrinkles using a 0 to 3 scoring system (4 classes) at both maximum frown and rest positions across several facial areas [1, 14, 15]. This framework also forms the basis of the Glabellar Line Scale (GLS), a global scoring method introduced by Kaufman‐Janette et al. [16].

In parallel, patient‐reported outcome measures based on questionnaires offer a complementary perspective by capturing subjective experiences of treatment effectiveness, satisfaction, and aesthetic improvement. Tools such as the Facial Line Satisfaction Questionnaire (FLSQ) [17, 18], Facial Line Outcome (FLO‐11) [19], FILLER‐Q, and FACE‐Q [18, 20] have been developed and validated to quantify these patient‐centered outcomes. While these instruments provide critical insights into individual perceptions, they are inherently subjective and difficult to standardize across populations or studies.

A third approach involves clinical assessments performed by trained investigators. The most prominent among these is the Global Aesthetic Improvement Scale (GAIS), a tool frequently used in clinical trials to offer an overall evaluation of aesthetic improvement [21, 22]. While such expert evaluations lend credibility through clinical judgment, they remain susceptible to inter‐rater variability and lack the precision of quantitative methods.

More recently, AI has emerged as a promising solution to advance the assessment of wrinkles. An example is the Standardized ColorFace AI‐based Wrinkle Assessment (SCAWA) scale based on Generative AI (Gen‐AI). Rather than real patient photographs, this scale relies on synthetic facial imagery generated by generative adversarial networks (Generative Adversarial Network (GAN)) [23]. AI‐driven systems offer the potential for objective, reproducible, and scalable evaluation, addressing many of the limitations associated with traditional manual and subjective methods. However, the performance and reliability of such systems are critically dependent on the quality and diversity of the data used during training. To achieve accurate and generalizable results, it is essential to train these models on high‐quality, unbiased datasets that represent a broad population spectrum encompassing different skin types, ages, and genders. Without such representativeness, AI tools risk producing biased or non‐representative outputs, limiting their clinical utility and fairness in real‐world applications.

Together, these diverse methods illustrate the complexity of wrinkle evaluation in aesthetic research and practice. The lack of methodological standardization continues to hinder consistent outcome reporting and comparative analysis. This fragmentation highlights the need for a unified, scientifically rigorous framework—such as AURA‐W—that leverages modern AI algorithms and provides broad applicability and precision to the field.

## 2 Objective

The objective of this study is to introduce a novel evaluation method for wrinkle assessment that combines advanced sensing technology with artificial intelligence. Our approach integrates a high‐resolution 3D imaging system with a consistent capture alignment, ensuring uniform lighting and stable head positioning. This reliable acquisition process enables precise and reproducible measurements of facial wrinkles. The data collected were then processed by an AI model trained to detect wrinkle lines and assign severity scores.

The study focuses on the comparison of patients treated with Botulinum Toxin, a therapy that has already been clinically proven to reduce wrinkles. Rather than validating the treatment itself, this work aims to demonstrate a new, objective, and reliable scoring system, AURA‐W, as a standard for evaluating treatment outcomes in aesthetic medicine.

### 2.1 Scope and Limitations

The present study was conducted as a single‐center investigation and involved volunteer patients recruited in Northern Italy. Consequently, the study population is geographically limited and biased with respect to gender, age, and ethnic background, which restricts the external validity and generalizability of the findings. In addition, the analysis was based on the assessments of a single physician and a single botulinum toxin formulation, further limiting the extent to which the results can be extrapolated to other clinical settings, evaluators, or treatment protocols.

Moreover, the study did not include a control group that did not undergo treatment, nor did it provide a systematic comparison of the AURA‐W assessment with other established computational or human‐based scoring methods. Such broader, multicenter, and comparative studies, involving more diverse patient populations, multiple evaluators, and different toxin formulations, were beyond the scope of the current work and will be addressed in future investigations.

## 3 Material and Methods

### 3.1 Measurement System

The Aura 3D Imaging system enables the detection and classification of facial wrinkles with high precision. It consists of 13 high‐resolution cameras and 18 light modules equipped with two sets of orthogonal polarization filters, allowing for comprehensive multi‐angle captures of the face and neck. The system reconstructs an accurate 3D model of the subject's face from multiple viewpoints.

To ensure consistency, the device includes a head‐positioning guidance system that helps the patient maintain a standardized pose relative to the cameras and lights during each session. This feature ensures that before‐and‐after images are captured from the same orientation and with the same light distribution, improving the reliability of the comparative analysis. Image acquisition is performed exclusively using the built‐in lighting of the system, making it independent of ambient light conditions. This controlled illumination ensures that the capture environment remains consistent across sessions, thereby enhancing the comparability of sequential scans.

The Aura 3D Imaging System incorporates Aura Unified Rating Algorithm (AURA), an AI‐driven framework for quantifying multiple cutaneous features. In this study, we focus on the wrinkle module, AURA‐W, for objective wrinkle analysis.

The wrinkles are highlighted on the user interface using a color scale ranging from green (score 1, indicating mild severity) to red (score 5, indicating grave severity).

**FIGURE 1:**
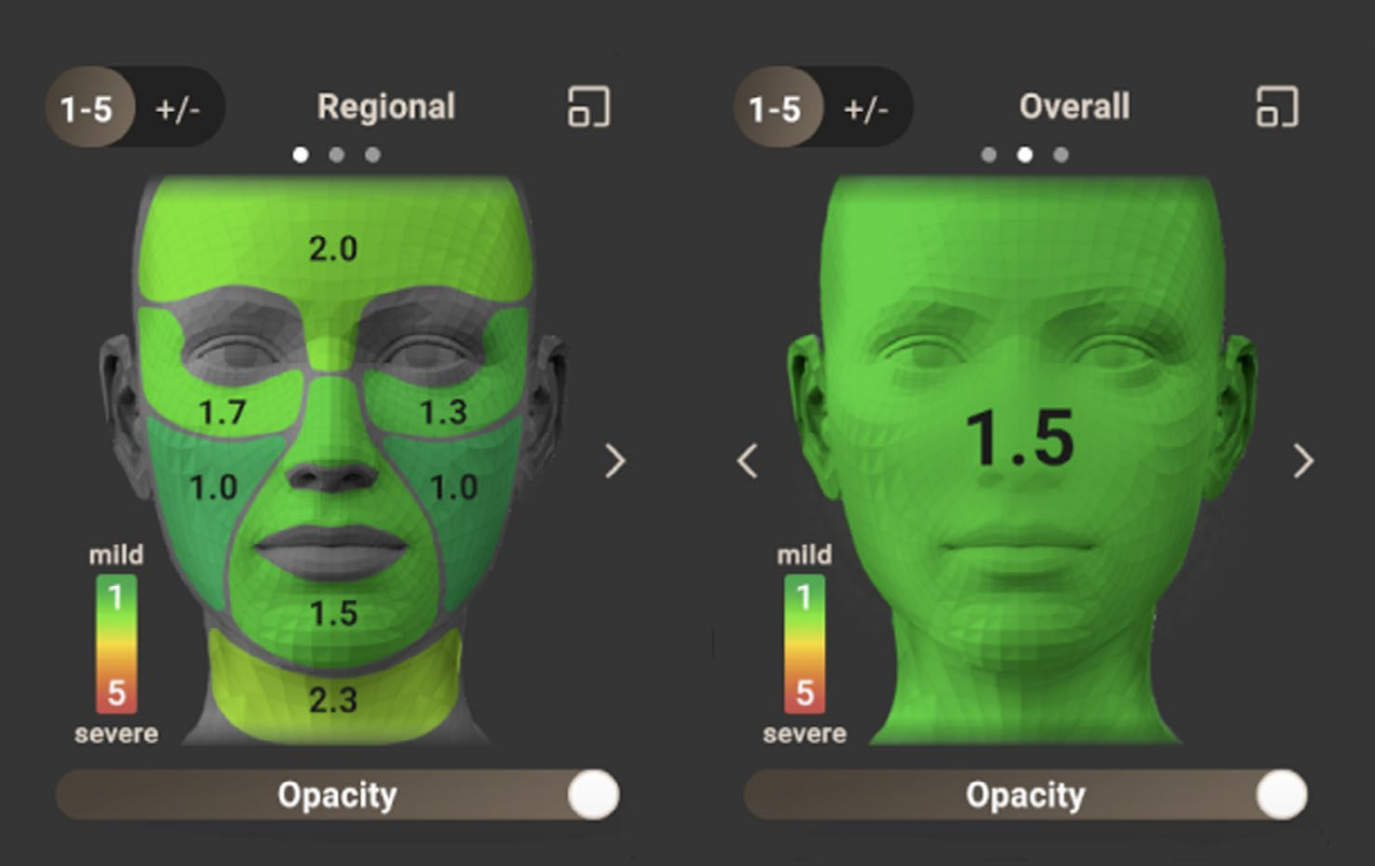
Visualization of regional wrinkle scores (left) and the global wrinkle score (right). Seven facial regions are individually scored: forehead and nose, mouth and nose, under‐eye right, under‐eye left, cheek right, cheek left, and neck.

The AURA‐W score is assigned both for specific facial regions and for the entire face. The defined regions are illustrated in Figure 1. On the left side of the figure, the scores are displayed for seven distinct regions: Forehead and nose, mouth and nose, under‐eye right, under‐eye left, cheek right, cheek left, and neck. On the right side, the visualization of the global score for the whole face is shown.

### 3.2 Aura Wrinkles Model

**FIGURE 2:**
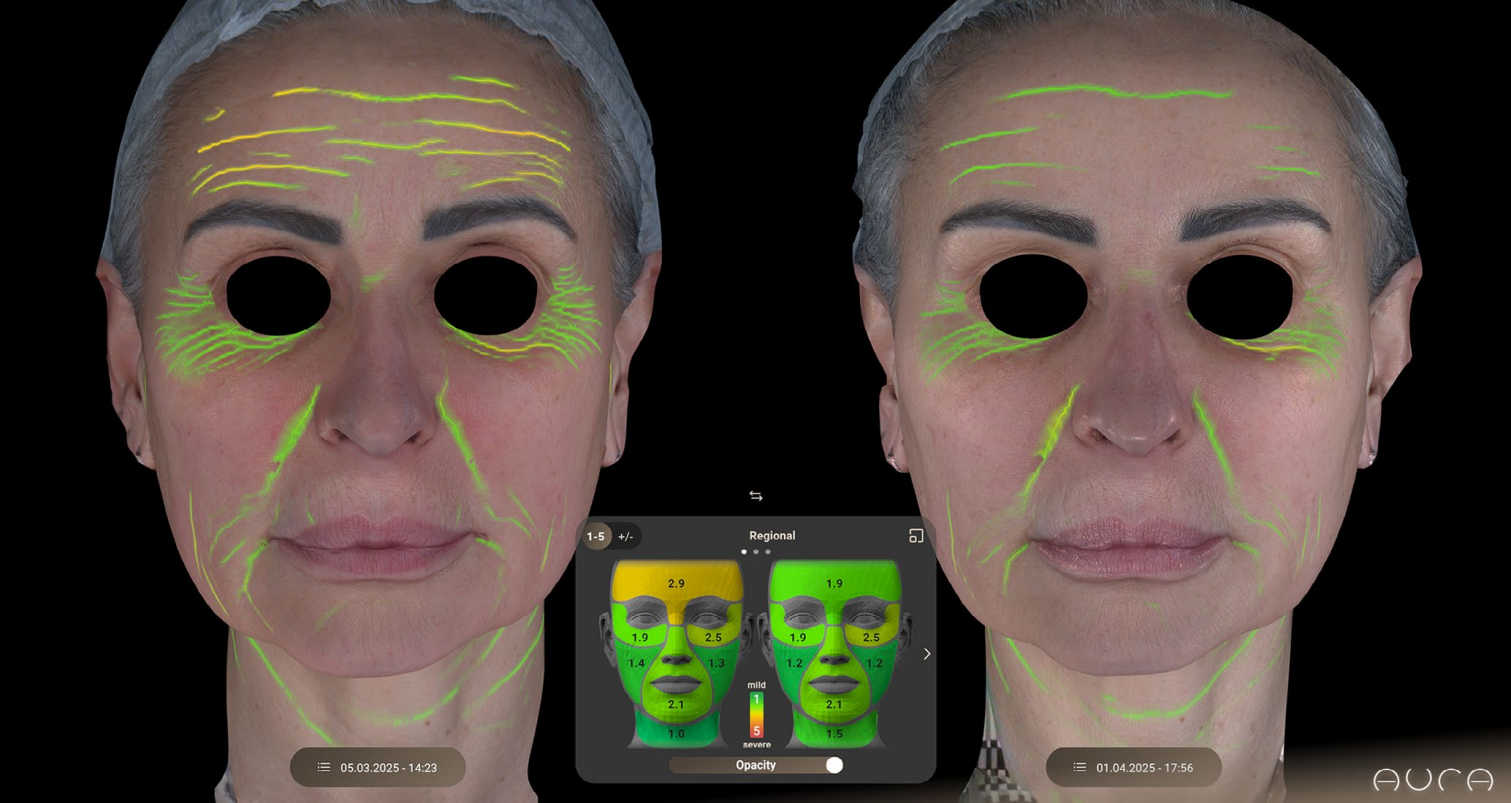
Visualization of wrinkle changes between the before and after captures and the corresponding regional scores. Wrinkles are color‐coded according to severity, ranging from 1 (green) to 5 (red).

Wrinkles are captured from multiple viewpoints and angles, and the final result is displayed overlaid on the 3D facial model. Wrinkle classification is performed using AI. The AI model provides a segmentation of the wrinkle lines and a corresponding score that reflects the severity of the wrinkle. Scores range from 1 to 5, where 1 indicates mild wrinkles and 5 indicates severe ones. The face is segmented into 7 areas, and each is scored individually. A compound score resulting from a weighted average of the regions is computed for the full face, see Figure 1. Additionally, the score is also visualized at the pixel level and represented via a color gradient from green (mild, 1) to red (severe, 5), Figure 2.

The model's scoring behavior is trained to align with the Lemperle Wrinkle Classification Scale [10]. For this study, to train the model, we collected a large and diverse dataset of over 3000 image sets from ∼1500 participants, including neutral and expressive facial images. The training data represents a wide range of skin types, genders, and ages, sourced from different regions of the world; see the Supporting Information for more details (Figures S1–S4). A committee of five aesthetic physicians annotated one‐third of the data and provided detailed labeling guidelines. The remaining images were annotated under strict quality‐assurance procedures and refined through several iterations with the committee to ensure high‐quality ground truth for training.

The annotated data were partitioned using a stratified sampling strategy based on age and skin type. Data were split into training, validation, and test sets, with 15% of the complete data set reserved for testing and 15% of the remaining data assigned to validation.

Multiple model architectures were evaluated for quality and performance. The selected model is a two‐stage design, generating separate segmentation and severity maps. The segmentation component classifies each pixel as belonging to a wrinkle or not, effectively delineating wrinkle lines from the rest of the facial surface. The score prediction is treated as a regression task, trained using a Mean Squared Error (MSE) loss function on a continuous target scale ranging from 1 to 5. The classification algorithm for wrinkle detection is based on a DeepLab architecture with a ResNet‐18 backbone, a well‐established combination in the Deep Learning (DL) community for image classification and segmentation tasks [24, 25, 26, 27]. Training was carried out over 44 epochs and demonstrated stable convergence. Additional technical details on the model and training data are available as Supporting Information accompanying the online article (Figures S5 and S6).

### 3.3 2D Vs. 3D Modalities

The AURA‐W score is computed from 2D image modalities, yet the full 3D nature of the capture pipeline plays a crucial role in achieving reliable and clinically meaningful results. The 3D component strengthens the method in several ways.

First, the training data inherently contains multiple views of the same wrinkle. This introduces a natural 3D consistency prior: The model learns that a wrinkle must appear coherent across viewpoints, even though the score itself is derived from 2D projections.

Second, model evaluation is performed in 3D. Any model that produces inconsistent wrinkle predictions across viewpoints is rejected. Only models that maintain stable geometry when reprojected into 3D space are accepted, enforcing a strong geometric constraint during validation.

Third, post‐processing is explicitly 3D‐aware. Wrinkle inference is refined on texture maps rather than flat 2D images, ensuring that the final representation aligns with the true surface geometry of the face.

Taken together, these steps make AURA‐W a genuinely 3D‐grounded metric. Although the final score is computed from 2D inputs, the system's reliance on 3D structure at every critical stage—training, validation, and post‐processing—provides robustness and accuracy that a purely 2D approach cannot match.

### 3.4 Botulinum Toxin

Injection of botulinum toxin is the most commonly performed nonsurgical cosmetic procedure worldwide in both women and men, and the number of injections increased by 35.5% between 2015 and 2019 [28].

This growing demand has been accompanied by the introduction of new formulations from multiple manufacturers [29]. Among these, Letibotulinum toxin A is a newly manufactured product derived from *Clostridium botulinum* strain CBFC26. Its safety and efficacy have been established in non‐cosmetic indications, including post‐stroke upper‐limb spasticity and dynamic equinus foot deformity in children with cerebral palsy [30, 31].

Furthermore, letibotulinumtoxin A has been shown to be as effective as onabotulinumtoxin A for the treatment of glabellar lines and crow's feet [32, 33, 34]. We selected this toxin specifically because of its recent market introduction to simulate a validation process using the Aura AURA‐W scale.

### 3.5 Study Design

Patients were enrolled according to predefined eligibility criteria, with inclusion and exclusion parameters presented in Table 2. All patients underwent digital acquisition with the Aura 3D Imaging System prior to treatment (T0). Follow‐up imaging was performed at T1, with a mean interval of 27.07 days after treatment. No additional aesthetic procedures (e.g., laser resurfacing, dermal fillers, chemical peels) were performed between T0 and T1 to avoid confounding effects.

**TABLE 2:** Detailed Inclusion and Exclusion Criteria.

**Inclusion criteria**
Subjects who meet all the following criteria are eligible for this study:
Aged between 18 and 75 years (inclusive)
Stable medical condition with no uncontrolled systemic disease
Female subjects of childbearing potential test negative for pregnancy and agree to use highly effective birth control during the course of the study
The before and after (BA) captures for a single procedure are acquired between 10 and 120 days apart
**Exclusion criteria**
Subjects who meet any of the following criteria are not eligible for this study:
Treatment with any serotype of botulinum toxin for any indication within the 12 months prior to screening, or any planned treatment with botulinum toxin of any serotype for any reason during the trial (other than the investigational treatment)
Known hypersensitivity to the study medication or its excipients
Any medical condition that may place the subject at increased risk due to exposure to botulinum toxin, including diagnosed myasthenia gravis, Eaton‐Lambert syndrome, amyotrophic lateral sclerosis, profound atrophy or weakness in the target muscles, or any other condition (at the investigator's discretion) that might interfere with neuromuscular function or contraindicate botulinum toxin therapy
Facial laser or light treatment, microdermabrasion, superficial peels, or retinoid therapy within the 3 months prior to screening or planned during the study
Apart from the procedures specified above, previous treatment with any facial aesthetic procedure in the glabellar area (including chemical peeling, injection with biodegradable fillers) within 12 months prior to screening or planned during the study
Previous insertion of permanent material in the glabellar area or planned during the study
Active skin disease/infection or irritation at the treatment area
Inability to substantially lessen glabellar frown lines even by physically spreading them apart
Use of a muscle relaxant within 2 weeks prior to screening or planned during the study
Pregnant, breastfeeding, or planning to become pregnant during the trial
Use of prohibited medication, including anticholinergic drugs, or drugs which could interfere with neuromuscular function, including aminoglycoside antibiotics and curare‐like compounds, within 2 weeks prior to screening or planned during the study
Planned surgery with general anesthetic (use of local anesthetic outside the glabellar area is permitted)
Chronic drug or alcohol abuse (as per the investigator discretion)

For the procedure, Letibotulinum Toxin A was injected at a dilution of 1.25 mL sodium chloride (NaCl), using a 1 mL Luer‐Lock (LL) syringe fitted with a TSK 33‐gauge (33G), 8 mm needle. A total dose of 50 units (U) of botulinum toxin was administered to the glabellar, forehead, and periorbital regions, with injection points adapted to each patient's individual anatomy and the dynamic facial expressions recorded during 3D imaging (Figure 4).

**FIGURE 3:**
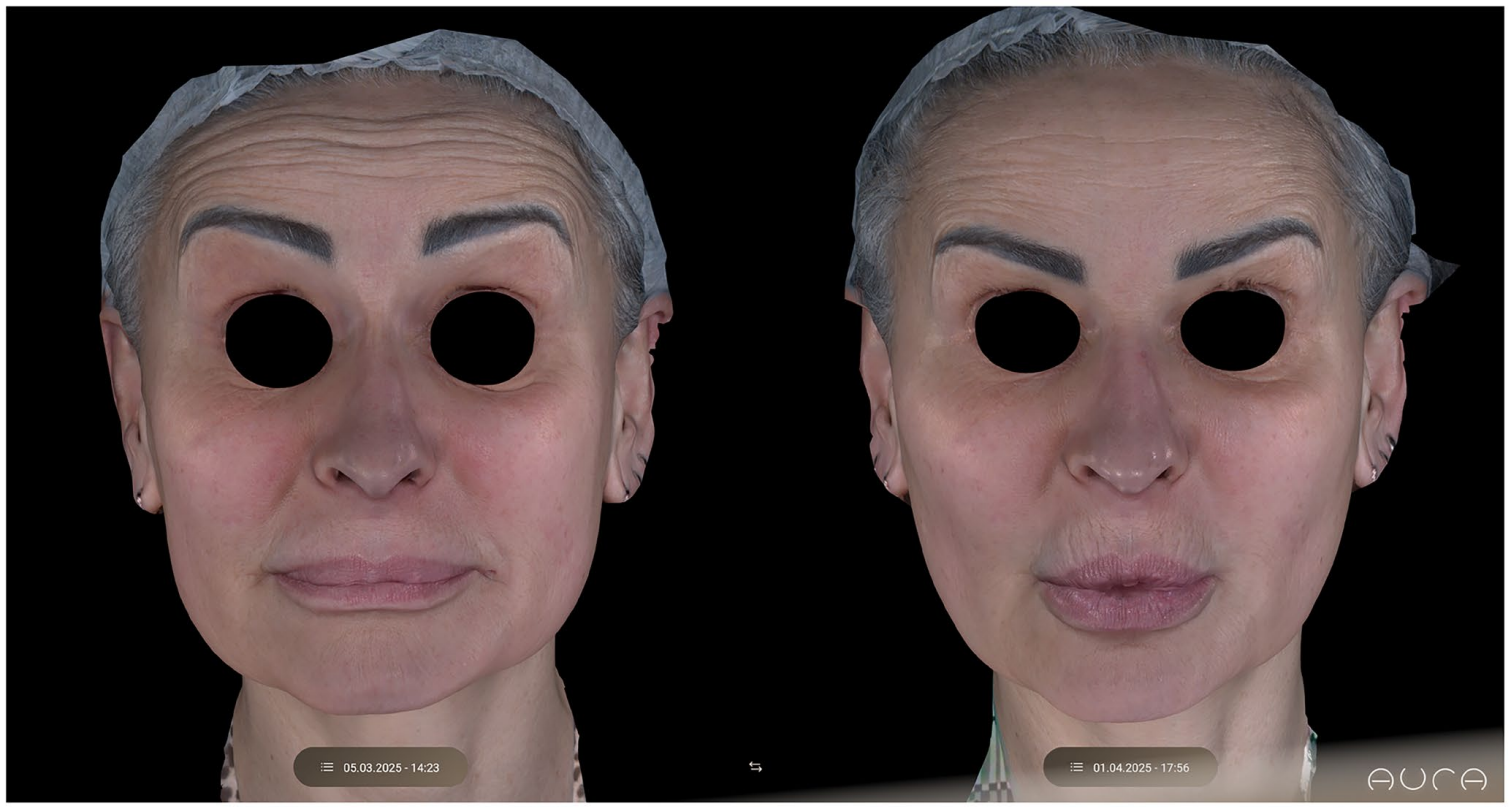
Visualization of wrinkle changes between the before and after captures during face expression (Surprise expression).

**FIGURE 4:**
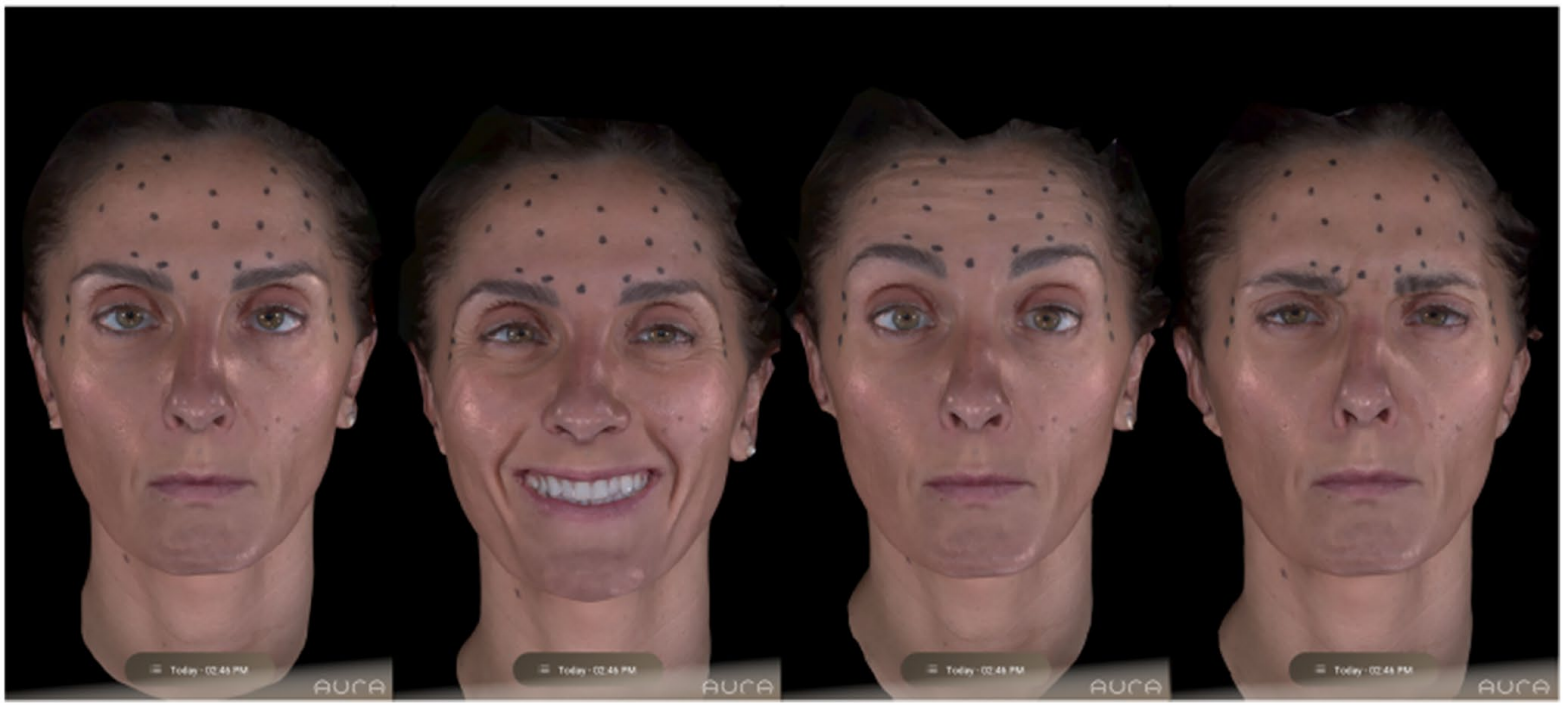
Injection sites for botulinum toxin, defined by the dot points marked on the patient's face. Images from left show neutral, smiling, surprised, and angry expressions. The deformation of the injection points with each expression illustrates how facial dynamics guide precise injection placement.

In addition to the capture of the face in a neutral expression, the Aura system allows the acquisition of four standardized facial expressions: Angry, smile, surprise, and kiss, see Figure 3 for an example. These expressions provide valuable information for guiding the identification of optimal injection sites and for evaluating treatment outcomes. As shown in Figure 4, the deformation of facial landmarks demonstrates how muscle activity influences the positioning of botulinum toxin applications. Each expression highlights specific regions of dynamic wrinkle formation: Smiling (“smile”) highlights crow's feet around the eyes; frowning (“angry”) highlights glabellar lines; raising the eyebrows (“surprise”) highlights horizontal forehead lines; and pursing the lips (“kiss”) highlights perioral or “smoker's” wrinkles. This dynamic analysis not only improves the precision of injection planning but also enables objective post‐treatment evaluation of therapeutic effectiveness across different facial regions.

### 3.6 Study Population

The study included a total of 100 subjects, comprising 88 women (88.0%) and 12 men (12.0%), with ages ranging from 25 to 75 years.

**FIGURE 5:**
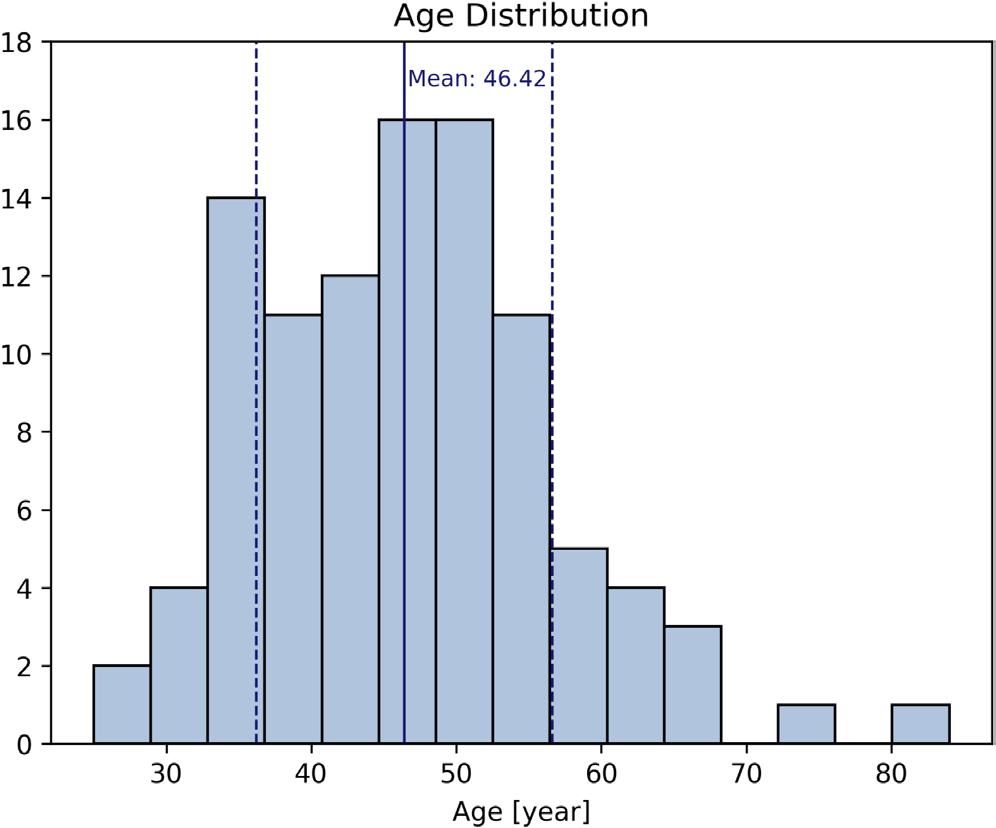
Age distribution of the population in the study, with a mean age of 46.42 years (SD = 10.21).

The average age of participants was 46.42 years, with a standard deviation of 10.21 years. A histogram of the age distribution is presented in Figure 5.

Of the 100 subjects enrolled, 80 met the inclusion criteria. One of the most stringent requirements for a valid BA analysis was the availability of paired captures acquired between 10 and 120 days apart. A total of 93 individual treatments were analyzed, since some subjects received more than one procedure. Among all the patients included in the study, 67 (84%) had only one procedure, while 13 patients (16%) had two procedures.

Each treatment instance was evaluated independently. In all cases, standardized imaging captures were obtained immediately before the injection (baseline) and again a few days after the procedure (post‐treatment), ensuring consistent assessment of the treatment effect.

**FIGURE 6:**
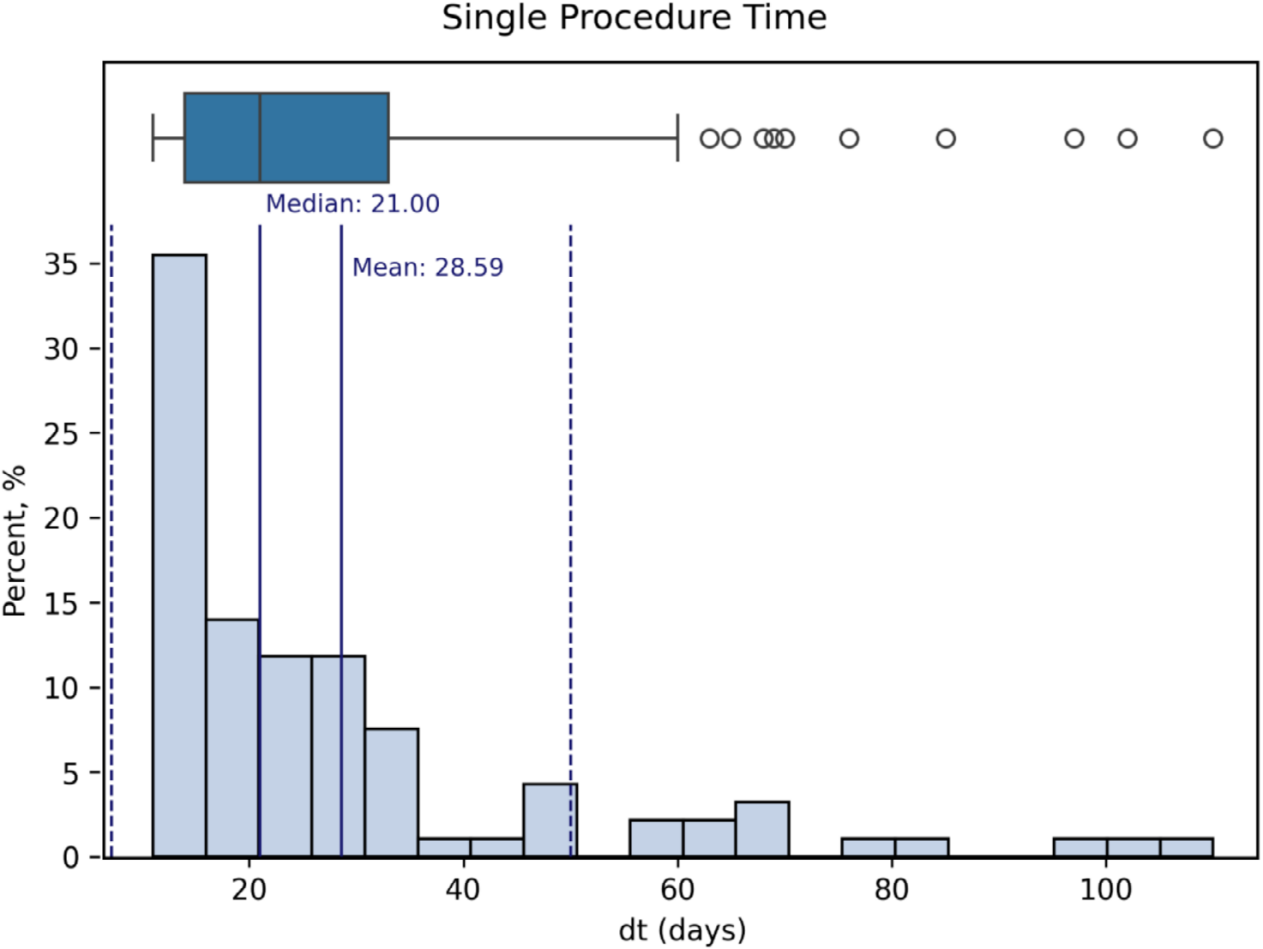
Distribution of the interval before and after the procedure, with a mean of 28.59 days (SD = 21.45) and a median of 21.00 days.

The distribution of the time interval (*dt*, in days) between the pre‐ and post‐treatment captures is shown in Figure 6. As seen in the figure, the majority of the BA images were taken approximately between 10 and 48 days apart, with an average of 28.59 days and a median of 21 days. This interval corresponds to the typical window in which the clinical effects of botulinum toxin become most apparent.

## 4 Results

A qualitative analysis of wrinkle score changes between before‐ and after‐treatment captures can be visualized and compared using the Aura app, as shown in the screenshot in Figure 2. While the wrinkles are highlighted and visible on the face, a score is assigned to each region in the bottom section.

To evaluate the effectiveness of the treatment across facial regions, we compared the difference in scores for BA treatment in the treated areas. According to our mask definitions, the treated areas correspond to Forehead and Nose for injections in the upper face, Under Eye Right and Under Eye Left for injections targeting the periorbital area, and Global for a composite score over the entire face.

**FIGURE 7:**
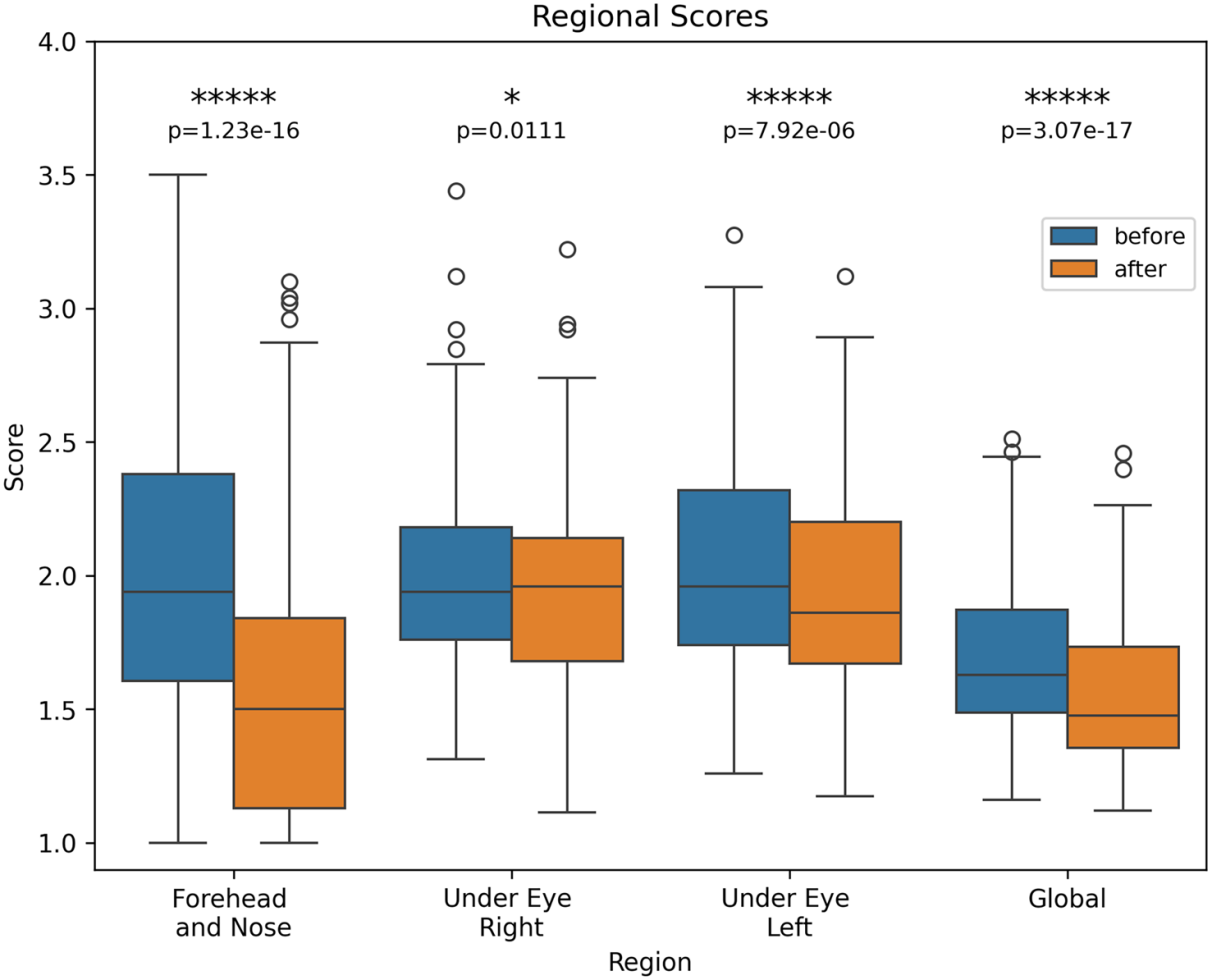
Distribution of the wrinkle scores for a single procedure before and after treatment for each treated area. Stars indicate significance difference between the two populations: *****p < 0.00001, ****p < 0.0001, ***p < 0.001, **p < 0.01, *p < 0.05.

Figure 7 presents box plots showing the distribution of scores before and after treatment for each region. Overall, the plots reveal a consistent reduction in posttreatment scores across all evaluated regions: Forehead and Nose, Under Eye Right, Under Eye Left, and Global.

To quantify the observed changes, we performed one‐sided paired *t*‐tests comparing pre‐ and post‐treatment scores. The one‐sided paired *t*‐test is a statistical method used to assess whether there is a significant directional difference between two related sets of measurements, in this case, scores recorded before and after treatment for the same subjects. Unlike a two‐sided test, which detects any difference between paired samples, the one‐sided test specifically evaluates whether the scores decreased following treatment. This is particularly appropriate in the context of wrinkle analysis after BA botulinum toxin treatment, where an improvement is expected to manifest as a reduction in the measured scores. By applying this directional test, we were able to assess whether the treatment led to statistically significant improvements in skin quality.

Prior to conducting the paired *t*‐tests, the normality assumption was evaluated on the paired differences (post–pre) for each analyzed region using the Shapiro–Wilk test (*n* = 93). Normality was not rejected for any region (Forehead and Nose: *p* = 0.0546; Under Eye Right: *p* = 0.4887; Under Eye Left: *p* = 0.2280; Global: *p* = 0.1707), supporting the use of the paired *t*‐test. All statistical tests were therefore performed as one‐sided analyses with the a priori directional hypothesis that post‐treatment scores are lower than pre‐treatment scores (H1: “after” < “before”).

Statistically significant reductions were observed in all regions. The most pronounced improvements occurred in the Global (*p* = 3.07 × 10–17) and Forehead and Nose (*p* = 1.23 × 10–16) regions, followed by Under Eye Left (*p* = 7.92 × 10–06) and Under Eye Right (*p* = 0.0111).

In the box plots, significance levels are indicated using a star notation based on *p*‐value thresholds defined in the caption of Figure 7. This visualization emphasizes the strength of statistical evidence supporting post‐treatment improvement, with * symbols highlighting the most significantly affected regions, particularly the Forehead and Nose and Global areas. The undereye zones also showed statistically significant improvements, though with smaller effect sizes.

The magnitude of the treatment effect varied across regions and can also be expressed as the percentage change from baseline, considering the status before treatment. The largest average percentage reduction in scores was observed for the Forehead and Nose region (−19.83%), followed by Global (−8.13%), while smaller improvements were recorded for Under Eye Left (−4.41%) and Under Eye Right (−2.24%).

**FIGURE 8:**
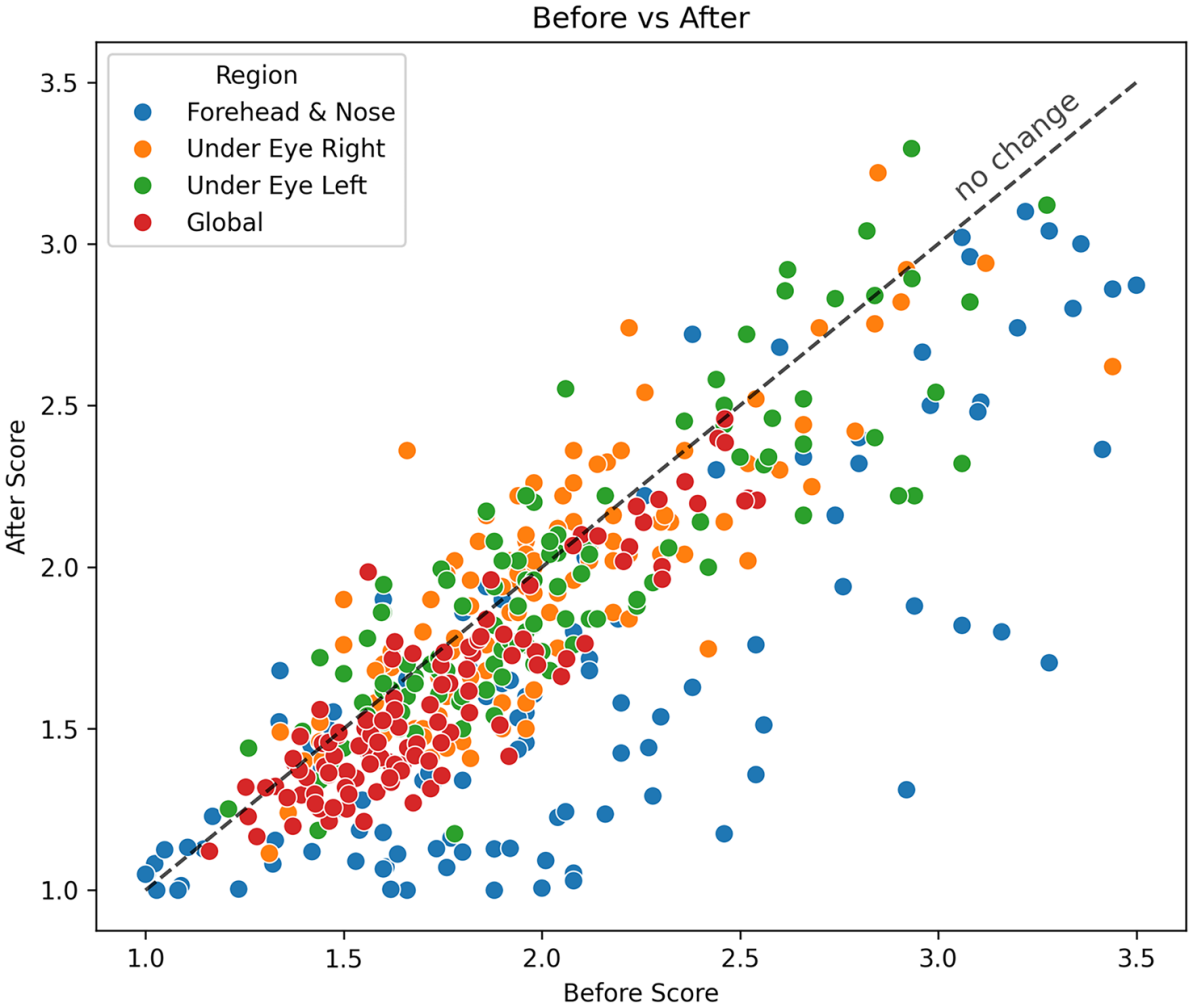
Comparison of wrinkle scores before and after treatment for each facial region. The dashed diagonal line represents the “no change” reference, where before‐ and after‐treatment scores are equal. Points below the line indicate a reduction in wrinkle score, while points above indicate an increase.

Figure 8 shows before–and–after comparisons of wrinkle scores for each facial region. Each point represents a paired measurement from a specific treatment session. The dashed diagonal line marks the “no change” reference, where before‐ and after‐treatment scores are equal. Points below the line indicate a reduction in wrinkle score following treatment, whereas points above indicate an increase. Most data points lie below the reference line, indicating overall improvement, with the effect being particularly pronounced in the Forehead and Nose region. This plot visualizes each individual data point collected during the study and confirms the results presented in Figure 7.

**FIGURE 9:**
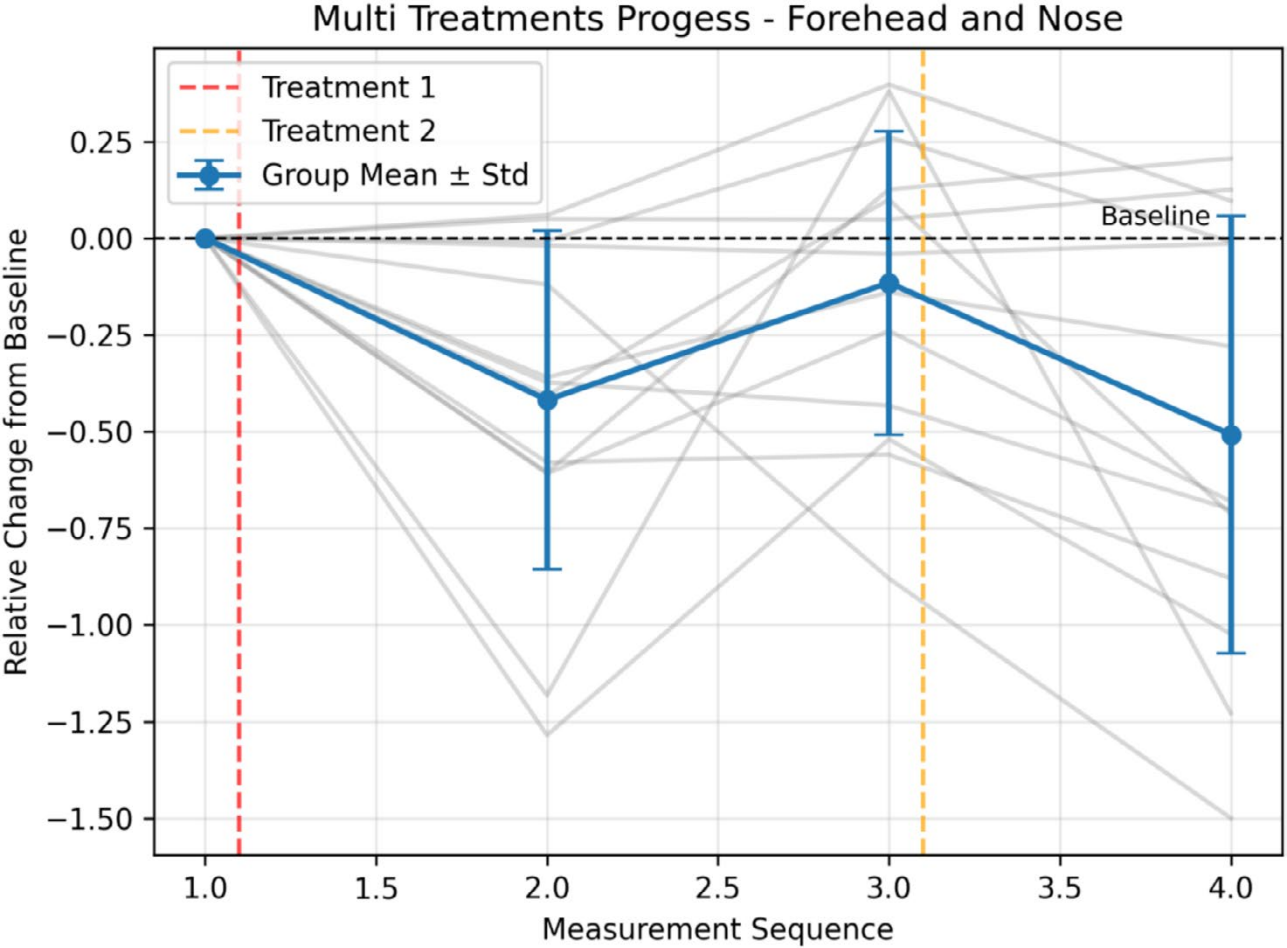
Progression over time of relative changes from baseline wrinkle scores in the Forehead & Nose. Light gray lines show individual patient trajectories, while the blue line represents the group mean ± standard deviation. Vertical dashed lines indicate the timing of the first (red) and second (orange) treatments.

It is possible to analyze the evolution of treatment effects over time. Only 13 participants received two treatment sessions, with an average interval of 276 days between the first capture and the last capture. Figure 9 shows the progression of relative changes from baseline wrinkle scores in the Forehead and Nose region across multiple measurement sessions.

Wrinkle scores in the *Forehead and Nose* region across multiple measurement sessions. Overall, most participants exhibited a reduction in wrinkle scores following each treatment, with values tending to increase again over time. Before the second treatment, wrinkle scores had partially returned toward baseline, followed by a further decrease after the subsequent treatment. This trend is clearly highlighted by the blue average line and is also visible in the trajectories of individual patients shown in light gray.

## 5 Discussion

The rationale of this study is to initiate the process of scientifically and reproducibly objectifying the effectiveness of botulinum toxin in reducing facial wrinkles.

While clinical trials with botulinum toxin have traditionally relied on subjective observational scales, the scientific literature demonstrates various objective assessment methods. Non‐visual objective approaches include [35] quantitative EMG analysis showing a significant decline in muscle activity following treatment, [36] three‐dimensional motion analysis systems that can objectively detect small changes in muscle tone during movement, and [37] acoustic radiation force impulse elastography for direct measurement of muscle stiffness changes. However, these methods assess muscle function rather than the aesthetic outcomes that are clinically relevant to patients and practitioners. For aesthetic applications, several objective visual assessment methods have emerged, including [38] the Antera 3D system for skin texture roughness evaluation and [39] Digital Analysis of Cutaneous Surface (DACS), providing three‐dimensional imaging and texture analysis. Despite their potential, these visual methods suffer from significant limitations, including low clinical adoption [40], poor repeatability for critical parameters such as wrinkle depth measurements, limited integration of AI for automated analysis and classification, and [41] lack of generalizability and standardization for clinical practice. Most studies employing these methods involve small cohorts of fewer than 30 participants, limiting statistical power and generalizability. Furthermore, a critical gap remains in the literature as none of these cited approaches propose a comprehensive standardized scale specifically designed for wrinkle measurement and classification. The Aura AURA‐W addresses these fundamental limitations by introducing a novel standardized scale and providing a comprehensive AI‐driven approach validated in a substantial cohort of 100 participants, combining high‐resolution 3D photogrammetry with automated artificial intelligence analysis to deliver standardized, reproducible, and clinically meaningful wrinkle assessment, establishing a new paradigm for objective evaluation in aesthetic botulinum toxin research.

The main limitations of this study still include its single‐center design, which reduces the ability of the software to provide broadly generalizable scores, as it is based on the results of a single physician and a single toxin formulation.

In addition, the study population consisted of volunteer patients recruited in Northern Italy, resulting in a sample that is biased in terms of gender, age, and ethnic background. These factors limit the generalizability of the findings to broader and more diverse populations.

It is anticipated that future multicenter studies, incorporating different molecules, a wider range of evaluators, and more heterogeneous patient cohorts, will address these limitations and further validate the scientific robustness and reproducibility of the software.

Another important limitation is the absence of a control group that did not undergo treatment, as well as the lack of a systematic comparison between the AURA‐W assessment and other established computational or human‐based scoring methods. While such comparative and controlled analyses would strengthen the interpretability of the results, they were beyond the scope of the present work and will be addressed in future investigations.

A further limitation of this study is that the follow‐up period was limited to one month. Consequently, the analysis primarily focuses on the pre‐ and post‐ treatment evaluation, emphasizing the potential of the proposed method as a new tool for objective assessment of treatment outcomes. Despite this short follow‐up, the approach demonstrates how a single standardized image capture can provide detailed evaluations across multiple aesthetic treatments. Although the AURA‐W results were positive across all areas analyzed, they were less evident in the periocular region. This is likely explained by the fact that the periocular analysis in this study encompassed a wider area that also included the tear trough, which is not typically targeted in botulinum toxin treatments. Thus, while the current findings are encouraging, future refinements that separate the periocular area from the tear trough are expected to yield even more precise outcomes.

As with all artificial intelligence systems, performance improves as larger datasets are analyzed. Therefore, it is reasonable to expect that the accuracy and reliability of this software will continue to increase. Nonetheless, the present findings already demonstrate a scientifically significant level of performance.

## 6 Conclusions

This study represents an initial step toward the integration of a scientific and reproducible wrinkle measurement system into botulinum toxin therapy. The AI‐powered software, trained by physicians during its development, is capable of recognizing wrinkles and assigning an objective score. Unlike traditional methods based solely on patient observation, the AURA‐W is acquired through a standardized and repeatable calculation and algorithm.

The findings indicate a reproducible reduction in wrinkle scores, with the most pronounced improvement in the *Forehead and Nose* region (−19.83%), followed by smaller changes in the Global and periocular areas. The primary goal of botulinum toxin therapy in the upper third of the face is the reduction of static mimic wrinkles caused by hyperspasticity of the facial muscles.

In the absence of an objective measurement tool, the evaluation of treatment efficacy has until now relied solely on clinical observation and subjective assessment. Such methods are inherently limited, prone to interobserver variability, and no longer adequate to meet the scientific and clinical standards required in modern aesthetic medicine.

## Author Contributions

F.V. and G.T. conceived and designed the study. F.V. collected the data. L.B. and G.T. performed the data analysis and interpretation. M.C. and E.B. contributed to the review of the manuscript and provided valuable feedback and domain expertise. All authors contributed to manuscript writing and revisions, and all authors have read and approved the final manuscript.

## Ethics Statement

This retrospective study was conducted in accordance with the Declaration of Helsinki. According to local regulations, formal ethical approval was not required, as the research involved analysis of anonymized clinical data obtained from routine aesthetic practice. All data were de‐identified before analysis, and patient confidentiality was maintained in compliance with GDPR regulations.

## Consent

Written informed consent was obtained from all patients for the use of their anonymized data and clinical photographs for research and publication purposes.

## Conflicts of Interest

Authors Gemma Taverni and Lukas Buchmann are employed at Hexagon Aura *Reality* AG.

## Supporting information


**Figure S1:** Age distribution of all data used for training the wrinkle detection model.
**Figure S2:** Facial expression distribution of all data used for training the wrinkle detection model.
**Figure S3:** Gender distribution of all data used for training the wrinkle detection model.
**Figure S4:** Skin type distribution of all data used for training the wrinkle detection model.
**Figure S5:** From left to right: Segmentation model prediction, the ground truth annotation, and the overlap of the predicted segmentation on the input image.
**Figure S6:** Global and regional scores for the same subject captured within a short timeframe. Dark blue is a mannequin manufactured by a VFX company, while the other series are two male and two female individuals.

## Data Availability

Research data are not shared.
