# Supplementary material for: Validation of AURA‐W: An AI‐Driven 3D Imaging System Metrics for Objective Wrinkle Assessment After Botulinum Toxin Treatment
Source: J Cosmet Dermatol. 2026 Jun 17;25(6):e70915. doi: 10.1111/jocd.70915 (PMC13273706; doi:10.1111/jocd.70915)
Supplement: Supplementary file 1 — Figure S1: Age distribution of all data used for training the wrinkle detection model. Figure S2: Facial expression distribution of all data used for training the wrinkle detection model. Figure S3: Gender distribution of all data used for training the wrinkle detection model. Figure S4: Skin type distribution of all data used for training the wrinkle detection model. Figure S5: From left to right: Segmentation model prediction, the ground truth annotation, and the overlap of the predicted segmentation on the input image. Figure S6: Global and regional scores for the same subject captured within a short timeframe. Dark blue is a mannequin manufactured by a VFX company, while the other series are two male and two female individuals. [file JOCD-25-e70915-s001.pdf]

SUPPLEMENTARY MATERIAL

Validation of AURA-W: An AI-Driven 3D Imaging System Metrics for Objective Wrinkle Assessment After Botulinum Toxin Treatment

1 | MODEL DETAILS

1.1 | Training Data

At the time of the clinical study to train and validate AURA-W skin scores, a series of data collection and labeling campaigns was executed. The training dataset is comprised of 3017 captures from 1443 individuals. The following plots summarize the distribution of the training data across key demographic and phenotypic variables, including age (Figure 1), facial expression (Figure 2), gender (Figure 3), and skin type (Figure 4). These visualizations provide an overview of the dataset composition and allow assessment of the balance and representativeness of the samples used to train the model.
Following data collection, labeling instructions were formulated in collaboration with five international expert dermatologists. In addition to informing the labeling instructions, these experts annotated and labeled 1000 images. Professional labelers annotated the capture photos of 2017 3D captures of 1443 individuals. Iterative trial rounds and labeling quality assurance tests were performed to ensure the precision of the instructions and the quality of annotation.
Multiple captures of the same participants within a short time-frame helped to ensure stability and reproducability of AURA-W scores.

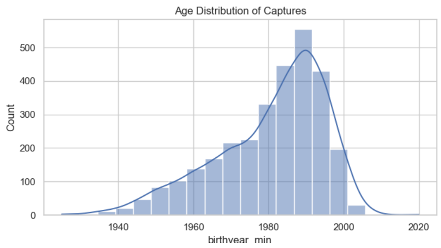

FIGURE 1 Age distribution of all data used for training the wrinkle detection model.

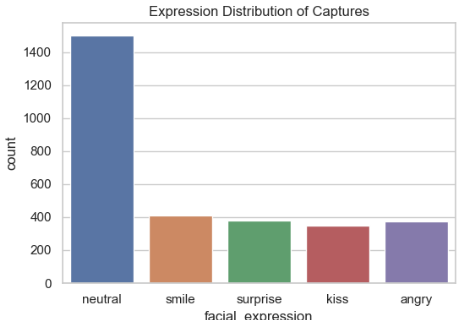

FIGURE 2 Facial expression distribution of all data used for training the wrinkle detection model.

1.2 | Model Design

Our segmentation model is trained on an annotated facial dataset and is used to segment different facial regions throughout all stages of the processing pipeline. The annotations comprise 17 semantic classes, including background and the following facial and contextual regions: ear, long beard, short beard, nose, eyeball, eyelashes, eyebrows, hair, whole face, person, eye, mouth, clothes, neck, under-chin region, and inside of the mouth. Model performance is monitored during training using the Intersection over Union (IoU) metric.

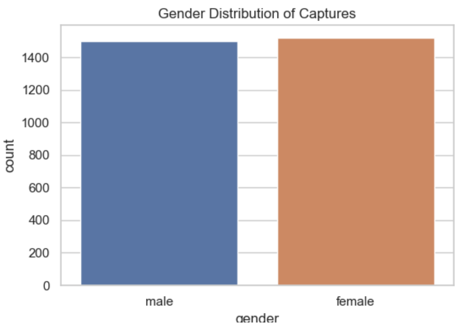

FIGURE 3 Gender distribution of all data used for training the wrinkle detection model.

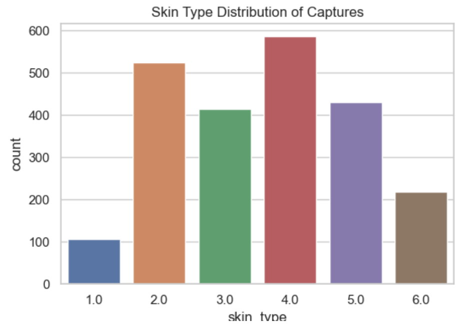

FIGURE 4 Skin type distribution of all data used for training the wrinkle detection model.

After 28 training epochs, the model converges with a mean IoU of 0.82 on the validation set and achieves a mean IoU of 0.78 on the held-out test set. Figure 5 illustrates qualitative segmentation results, showing the model prediction, the ground truth annotation, and the overlap of the predicted segmentation on the input image from left to right.

For AURA-W, we employed a custom loss function for wrinkle detection and severity score classification, designed to evaluate model predictions exclusively on ground-truth wrinkle pixels, thereby focusing the optimization process on clinically relevant regions. This targeted loss formulation reduces the influence of background and non-wrinkle areas, leading to more accurate learning of wrinkle-specific features and severity levels.

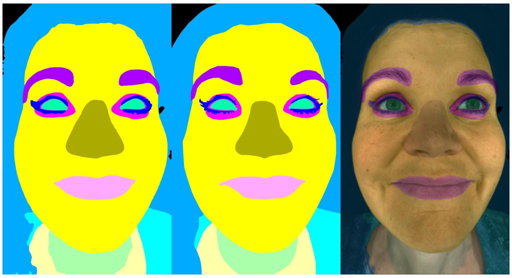

FIGURE 5 From left to right: segmentation model prediction, the ground truth annotation, and the overlap of the predicted segmentation on the input image.

All models were trained on an NVIDIA Tesla V100 GPU for 44 epochs, ensuring stable convergence and efficient optimization.

The following model backbones were considered :

- ResNet50
- MobileNet
- ResNet34
- ResNet18
- ResNet18 + pre-processing.

The best overall results were obtained using a ResNet18 backbone with customized pre-processing.

1.3 | Model and Score validation

The AI model was validated on the validation set and the scoring algorithm that assigns scores to the different areas in the face and the global score was validated independently. Different captures of the same individual will always have some variation in their segmentation and the assignment of a particular part of the facial surface to the semantic scoring areas will also differ. Additionally, slight variations in the individuals facial expression, environment lighting conditions, skin conditions and hair arrangement will lead to noise in the inferred wrinkle scores. To validate the scores, 4 individuals and a specifically manufactured face mannequin were captured within a short timeframe and the scores

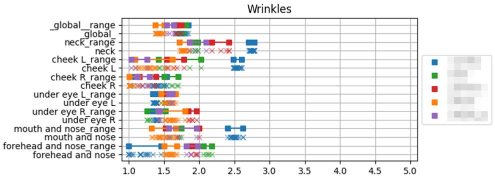

FIGURE 6 Global and regional scores for the same subject captured within a short timeframe. Dark blue is a mannequin manufactured by a VFX company, while the other series are two male and two female individuals.

compared, see Figure 6.
